# Testicular Busulfan Injection in Mice to Prepare Recipients for Spermatogonial Stem Cell Transplantation Is Safe and Non-Toxic

**DOI:** 10.1371/journal.pone.0148388

**Published:** 2016-02-12

**Authors:** YuSheng Qin, Ling Liu, YaNan He, Chen Wang, MingYuan Liang, XiaoLi Chen, HaiSheng Hao, Tong Qin, XueMing Zhao, Dong Wang

**Affiliations:** 1 The Key Laboratory for Farm Animal Genetic Resources and Utilization of Ministry of Agriculture of China, Institute of Animal Science, Chinese Academy of Agriculture Sciences, Beijing, 100193, China; 2 College of Animal Science and Technology, Jilin Agriculture University, Changchun, 130118, China; 3 Guangxi University, Nanning, 530004, China; Inserm, FRANCE

## Abstract

Current methods of administering busulfan to remove the endogenous germ cells cause hematopoietic toxicity, require special instruments and a narrow transplantation time. We use a direct testicular injection of busulfan method for preparing recipients for SSC transplantation. Male ICR mice (recipients) were divided into four groups, and two experimental groups were treated with a bilateral testicular injection of 4 or 6 mg/kg/side busulfan (n = 60 per concentration group). Mice received an intraperitoneal injection (i.p.) of 40 mg/kg busulfan (n = 60, positive control) and bilateral testicular injections of 50% DMSO (n = 60, negative control). Donor SSCs from RFP-transgenic C57BL/6J mice were introduced into the seminiferous tubules of each recipient testis via efferent duct injection on day 16–17 after busulfan treatment. Recipient mice mated with mature female ICR mice and the number of progeny was recorded. The index detected at day 14, 21, 28, 35 and 70 after busulfan treatment. Blood analysis shows that the toxicity of busulfan treated groups was much lower than i.p. injection groups. Fertility was restored in mice treated with busulfan and donor-derived offspring were obtained after SSC transplantation. Our study indicated that intratesticular injection busulfan for the preparation of recipients in mice is safe and feasible.

## Introduction

The self-renewal and differentiation of spermatogonial stem cells (SSCs) is the foundation of spermatogenesis [1]. The mechanisms of spermatogenesis, sperm deformation and the maturation of sperm in humans have not been fully elucidated [2, 3]. Furthermore, male infertility that resulted from spermatic deformities and abnormal spermatogenesis, amongst other causes, are becoming increasingly common [4–6]. In the background of a high incidence of hereditary diseases worldwide [7], it is necessary to intensively research the mechanisms of male spermatogenesis to explore male contraception techniques and other birth control technologies, which have become the focus of male reproductive research field. However, the lack of adequate experimental animal models and experimental approaches has delayed progress in this field.

SSCs are self-renewing and can maintain fertility through the lifetime of male individuals. The success of xenograft technology provides a new method to develop an experimental animal model and extend the possible experimental approaches [8]. Transplanting xenograft testis cell into recipient testes, such as mice would enable donor germ cell colonize [9–11], and even generated spermatogenesis [12], not only for clinical medicine applications, but for the theoretical study and manipulation of human spermatogenesis.

Recipient preparation is the key step for SSC transplantation: the endogenous germ cells need to be removed to make space for exogenous SSCs [13]. Busulfan is an alkylating agent, which causes the apoptosis germ cell populations [14]. The method of administering busulfan through an intraperitoneal (i.p.) injection has been developed and used extensively to prepare recipients for SSC transplantation [15–17]. However, administering busulfan through an i.p. injection in rodents, can also inhibit hematopoiesis and cause severe, and sometimes lethal, side effects [18]. Therefore, the irradiation method was developed and has shown some promising results in rats [19], mice [20], rhesus monkeys [21], and goats [22]; but this approach needs specialized and expensive instruments, and leads to calcification in the seminiferous tubules [23]. A heat shock method has also been used in mice [24], but the narrow transplantation window restricts its extensive use.

In order to set up a safe, feasible and low cost recipient preparation method, we directly injected busulfan into the recipient testes and obtained the donor-derived offspring after SSC transplantation in our previous study [25]. The most important factor with this method, was that no mouse death was resulted during recipient preparation. When busulfan was injected into the target organ testis, we deduced that most of the busulfan acted directly on the endogenous germ cells and very little of it permeated the blood circulation and was transferred into other organs. However, the toxic effects of administering busulfan through a testis injection are unknown, which is a question we sought to answer in this study. In order to illustrate the detailed impact of busulfan on recipients after a testis injection, we analyzed physiological variations in recipient mice, which will help us to optimize the injection dose of busulfan and the timing for SSC transplantation when this method is used.

## Materials and Methods

### Animals

Sexually mature 6-week-old ICR male mice (Beijing Vital River Laboratories, Beijing, China) were used as SSCs transplantation recipients. Transgenic C57BL/6J male mice expressing red fluorescent protein (RFP) were purchased from the Chinese Academy of Medical Sciences (CAMS, Beijing, China) and used as donors. RFP is expressed by the DsRed-expressing gene which is driven by the chicken β-actin promoter and several tissues, including the testes, in transgenic mice exhibit red fluorescence under excitation by ultraviolet (UV) light. Sexually mature ICR female mice (Beijing Vital River Laboratories, Beijing) were used as mating partners for the recipients. All the mice were housed at 22–25°C with natural light/dark cycles; food and water were available ad libitum. During experiment, we used humane endpoints and euthanized animals prior to the end of our experiments. Physical state of all the mice was observed five times in a day (07:00, 11:00, 15:00, 19:00 and 23:00) beginning at 0 hour after busulfan treatment, which continued until the end of experiments. Pain reliever was administered immediately when any clinical or toxicological symptoms were showed, and euthanasia (Cervical dislocation) was performed if the mice showing abnormal breathing or abnormal locomotion, head tilt, lethargy or paresis. In the study, animal care and samples collection procedures were approved and conducted under established standard of the Institute of Animal Science, Chinese Academy of Agricultural Sciences, Beijing, China. Mice were housed and prepared for transplantation in accordance with the Experimental Animals Standard Assembly (ISBN 9787506664486) of Institute of Laboratory Animal Science, Chinese Academy of Medical Sciences (CAMS), and all procedures used in the present study were approved by CAMS.

### Recipient treatment and transplantation

ICR male mice, used as recipients, were randomly allocated into four groups: the i.p. group (60 mice, positive control), two experimental groups (60 mice for each testis injection group) and the negative control group. Mice in the i.p. group (positive control) received i.p. injections of 40 mg/kg busulfan and negative control group mice received bilateral testicular injection of 50% dimethyl sulfoxide (DMSO; Sigma, St Louis, MO, USA), respectively. Mice in the two experimental groups were administered with either 4 or 6 mg/kg/side busulfan in each testis according to the protocol [25]. Briefly, the mice were anaesthetised, the testes were gently squeezed into the scrotum from the abdominal cavity and the scrotum was then disinfected with rubbing alcohol. Next, the testis was fixed between the left forefinger and thumb so that the longitudinal axis of the testis was parallel to the left forefinger and thumb with the testicular tail outside the body. The testis was then injected from the testicular tail along the long axis of the testis. Busulfan was injected directly into the testis using a sterile infant transfusion needle held with the right hand. Before the injections, busulfan (Sigma) was dissolved in DMSO, and diluted in distilled water to final concentrations of 4, 6 and 40 mg/mL. Mice were anesthetized with 4% pentobarbital sodium (Merck, Darmstadt, Germany) by i.p. injection at a dose of 60 mg/kg before busulfan or DMSO were administered.

Donor testis cell suspensions were prepared using testes from the offspring of RFP-transgenic C57BL/6J mice at 4–7 days after birth. Briefly, the seminiferous tubules of donor testes were digested with a two-step enzymatic digestion [13]. The testicular cells were centrifuged at 650*g* for 5 min at room temperature, and the supernatant removed. The cell pellets were suspended in Dulbecco’s PBS supplemented with 5% fetal bovine serum. After being cultured for 2h in 0.2% gelatin at a concentration of 2 × 10^7^ cells/mL, about 10 μL of the suspension was introduced into the seminiferous tubules of each recipient testis via efferent duct injection on day 16–17 after busulfan treatment.

### Analysis of fertility in the recipients after busulfan treatment

The recipient mice naturally mated with sexually mature ICR female mice after busulfan treatment at predetermined time, and each recipient mated with two ICR female mice each time. Information was recorded regarding the recipients and their progeny such as identity number, offspring number in each nest and offspring gender. There was only one record of a mating result in the negative control group.

### Routine blood analysis

After busulfan or DMSO treatment, peripheral blood samples were collected from the orbital sinus of five anesthetized recipient mice at days 14, 21, 28, 35 and 70, according to Hoff’s method [26]. Blood samples of approximately 30 μL were collected from each mouse in 2 mL diluent (MEK-6318K, Nihon Kohden, Japan) to prevent the blood from clotting at room temperature. The blood samples were used for the analysis of red blood cells (RBC), white blood cells (WBC), hemoglobin (HGB) and platelets (PLT) with an Automatic Hematology Analyzer MEK-6318K (Nihon Kohden, Tokyo, Japan).

### Detecting cavity diameter of the seminiferous tubules

After blood samples were collected, two recipient mice were randomly selected from each group and sacrificed. The bilateral testicles were then fixed in neutral formalin solution at 4°C for 7 days, embedded in paraffin, then cut into 4–5 μm thick sections and stained with Hematoxylin and Eosin. Three non-adjacent sections per half testis were selected to illustrate the variation process of the seminiferous tubules under a light microscope (Olympus IX71, Tokyo, Japan). The luminal diameters of the round seminiferous tubules cross-sections measured after busulfan treatment and analyzed by Image-ProPlus 6.0 software.

### Body weight and testis weight analysis

Five mice were randomly selected from each of the groups on days 14, 21, 28, 35, and 70 after treatment. After euthanasia, the mice were weighed, and both testes were collected and weighted using a Precision Electronic Scale (ES320; D&T, Tianjin, China).

### Statistical analyses

In order to detect whether the groups demonstrate equivalent variance in one parameter, we used the Kruskal-Wallis one way analysis of variance by SAS package software (SAS 8.01 software; SAS Institute Inc. Cary, NC, USA) to assess the overall effects.

## Results

### Testes weight and body weight

**Fig 1:**
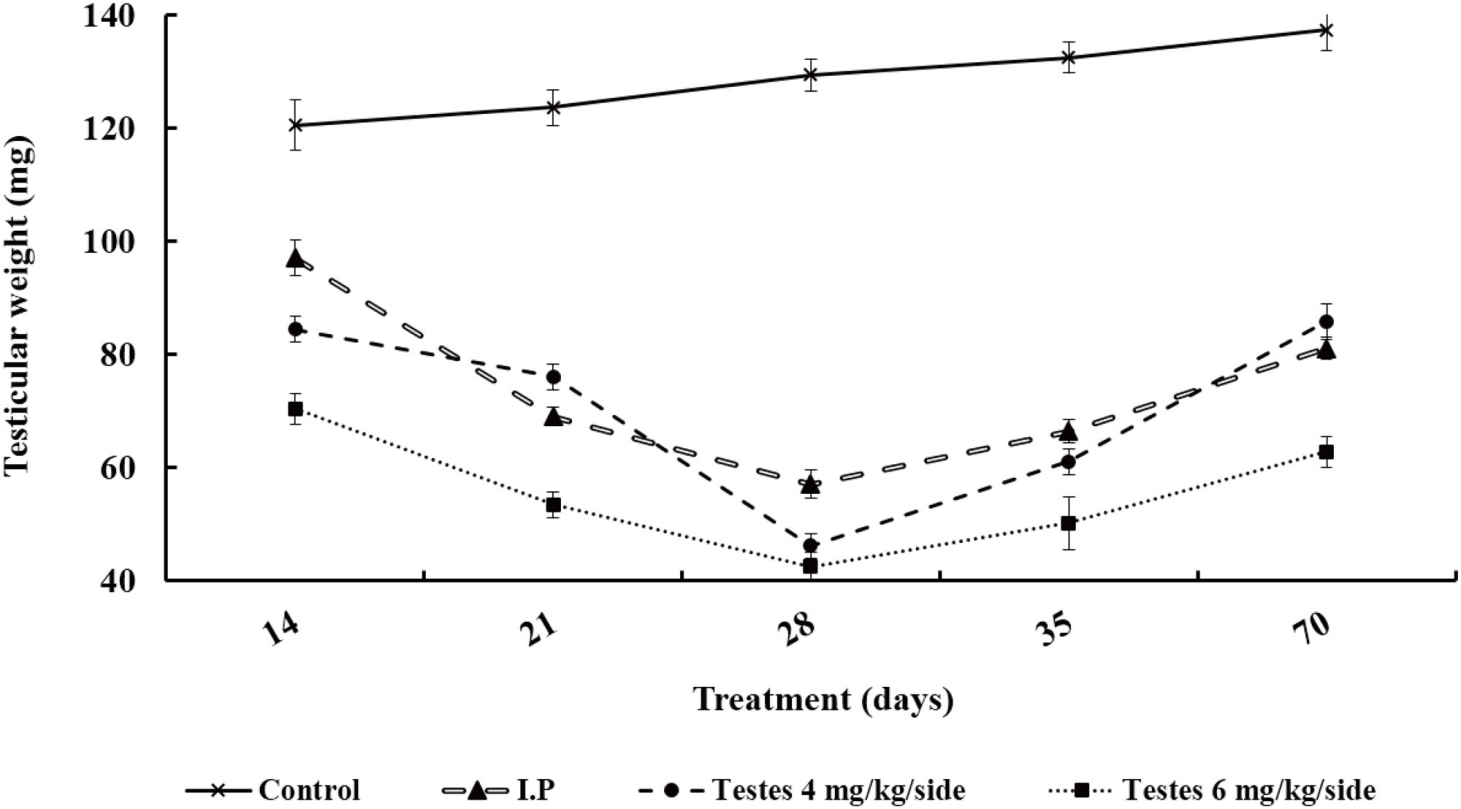
The change of testis weight after busulfan treatment over time.

**Fig 2:**
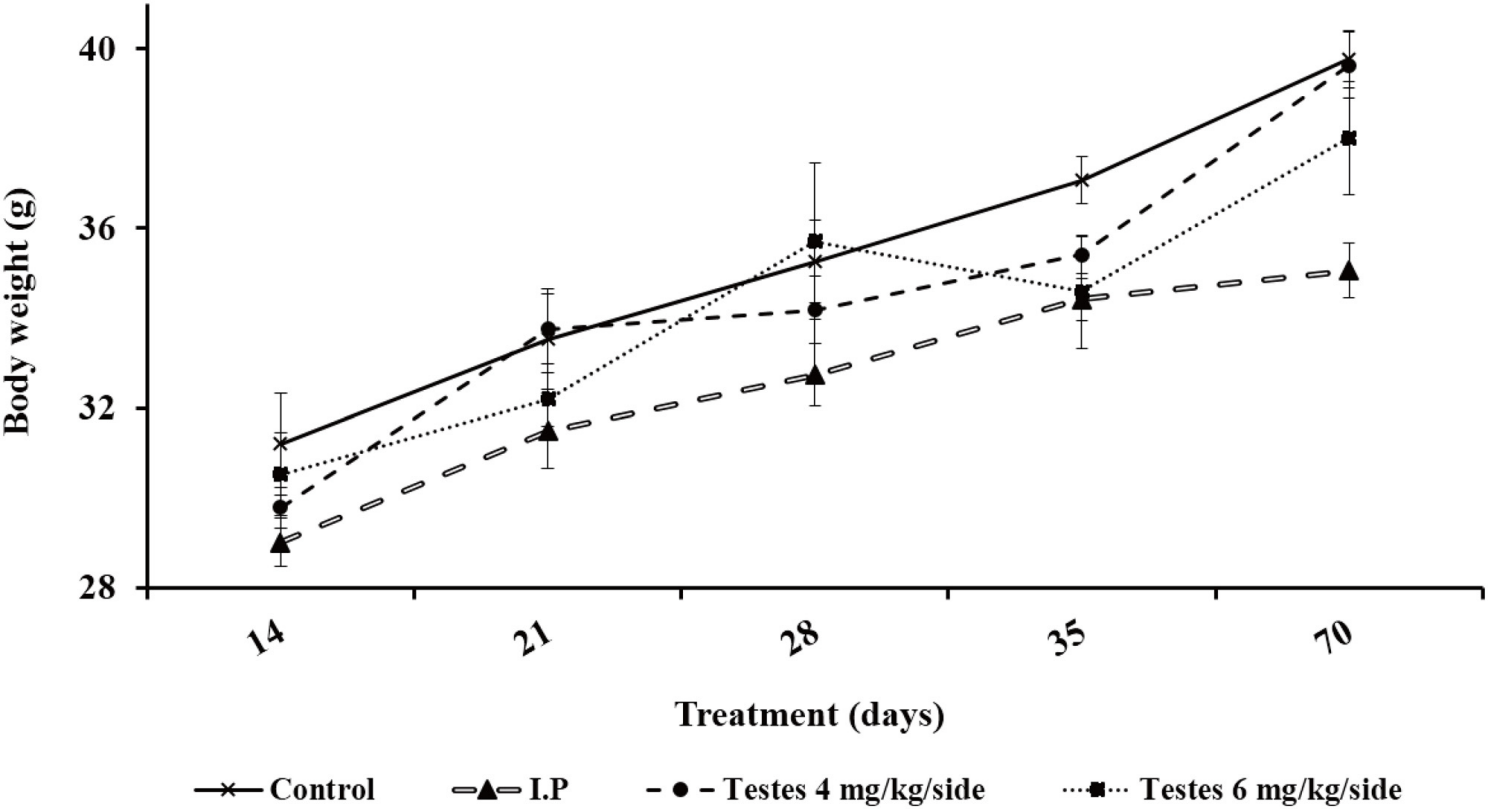
The change of body weight after busulfan treatment over time.

The weights of testes decreased slowly in both the testis and i.p. injection groups from day 14 after busulfan treatment, reaching the lowest level on day 28 after busulfan treatment before gradually increasing. None of the groups were restored to the level of the control group by day 70 (Fig 1 and Table 1). The average testicular weight of mice in the 6 mg/kg/side group was always lower than that of mice in the 4 mg/kg/side group or the i.p. injection group. The average body weight increased to an almost constant level until day 70, and there were no significant differences between i.p. injection group and testis injection group except on day 70 (*P* > 0.05) (Fig 2 and Table 1).

**Table 1:** The change of body weight and testes weight after busulfan treatment.

Day after treatment	Group	Bodyweight (g)	Testicular weight (mg)
14	Control	31.19±1.13 ^a^	120.58±4.44 ^a^
	I.P.	29.00±0.53 ^a^	97.14±3.11 ^b^
	Testes 4	29.79±0.45 ^a^	84.52±2.27 ^c^
	Testes 6	30.53±0.93 ^a^	70.44±2.77 ^d^
21	Control	33.53±1.13 ^a^	123.68±3.21 ^a^
	I.P.	31.49±0.83 ^a^	69.10±1.59 ^c^
	Testes 4	33.75±0.78 ^a^	76.12±2.32 ^b^
	Testes 6	32.19±0.60 ^a^	53.50±2.28 ^d^
28	Control	35.25±0.92 ^a^	129.46±2.84 ^a^
	I.P.	32.73±0.69 ^a^	57.10±2.55 ^b^
	Testes 4	34.18±0.76^a^	46.20±2.24 ^c^
	Testes 6	35.70±1.74 ^a^	42.56±2.52 ^c^
35	Control	37.07±0.52^a^	132.54±2.79 ^a^
	I.P.	34.42±0.47^b^	66.46±2.10 ^b^
	Testes 4	35.41±0.41^b^	61.14±2.27 ^bc^
	Testes 6	34.58±1.26^b^	50.20±4.77 ^c^
70	Control	39.76±0.64 ^a^	137.40±3.64 ^a^
	I.P.	35.04±0.61^b^	81.10±1.95 ^b^
	Testes 4	39.63±0.73^a^	85.90±3.20 ^b^
	Testes 6	38.01±1.26 ^ab^	62.86±2.75 ^c^

Testes 6, testis 6 mg/kg/side dose group; testes 4, testes 4 mg/kg/side dose group; I.P., intraperitoneal busulfan injection group; Control, negative control group. Different letters (a, b, c and d) within a column denote a statistically significant difference compared with i.p. injection group (P < 0.05).

### Detection of luminal diameter of the seminiferous tubules

**Fig 3:**
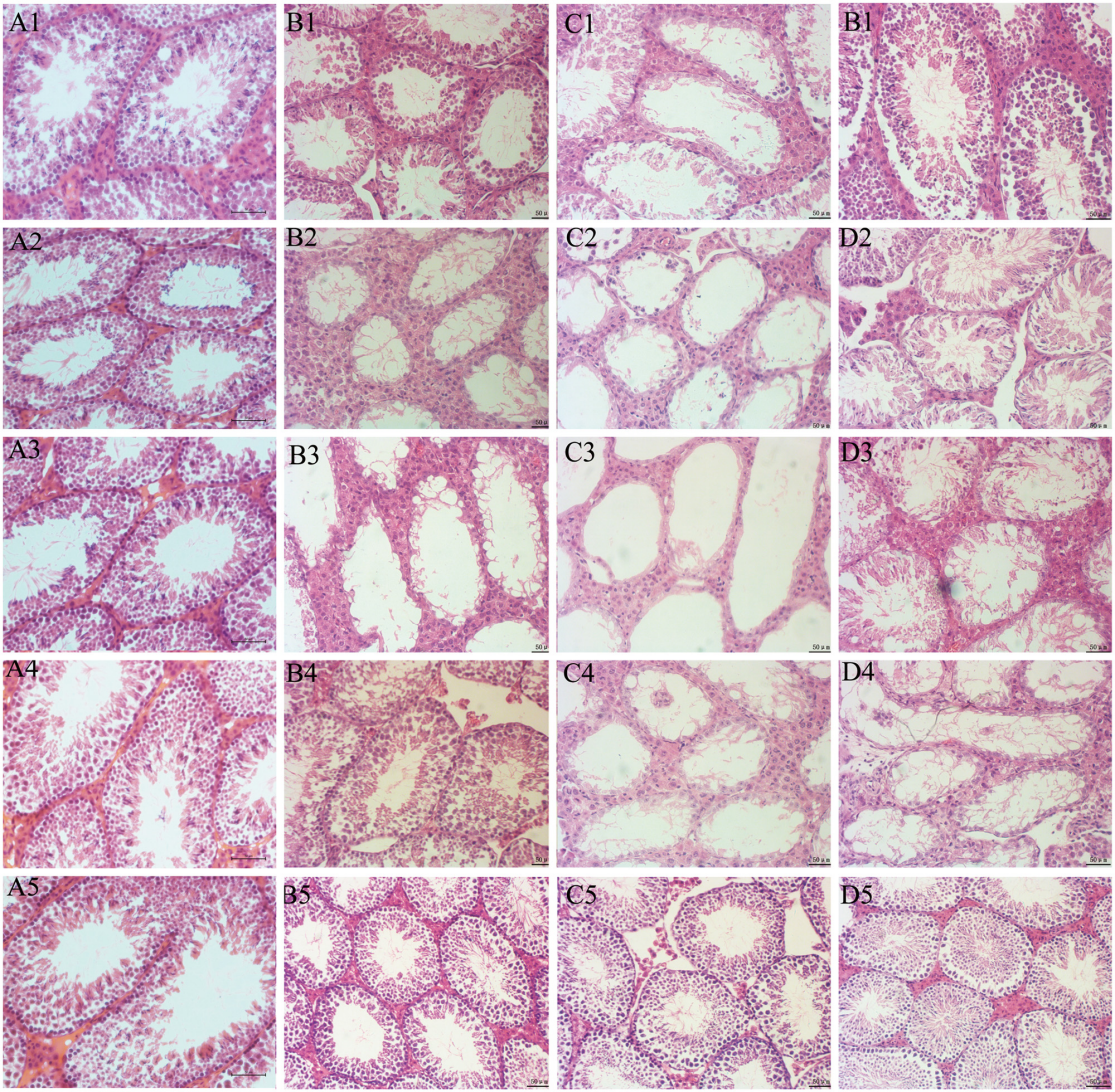
Histological sections of testicular tissue taken from mice in the four groups at different time points. Hematoxylin & Eosin staining is shown. A-D Numbers 1–5 refer to the day after treatment on which the samples were taken, where 1 = day 14, 2 = day 21, 3 = day 28, 4 = day 35 and 5 = day 70. A1–A5) The negative control group mice were treated with 50% DMSO at corresponding time points. B1–B5) Testis tissue sections from mice in the 4 mg/kg/side testis-treated group at corresponding time points. C1–C5) Testis tissue sections from mice in the 6 mg/kg/side testis-treated group at corresponding time points. D1–D5) Testis tissue sections from mice in the 40 mg/kg I.P. injection group. Scale bars(A = 100μm, B,C,D = 50 μm).

**Fig 4:**
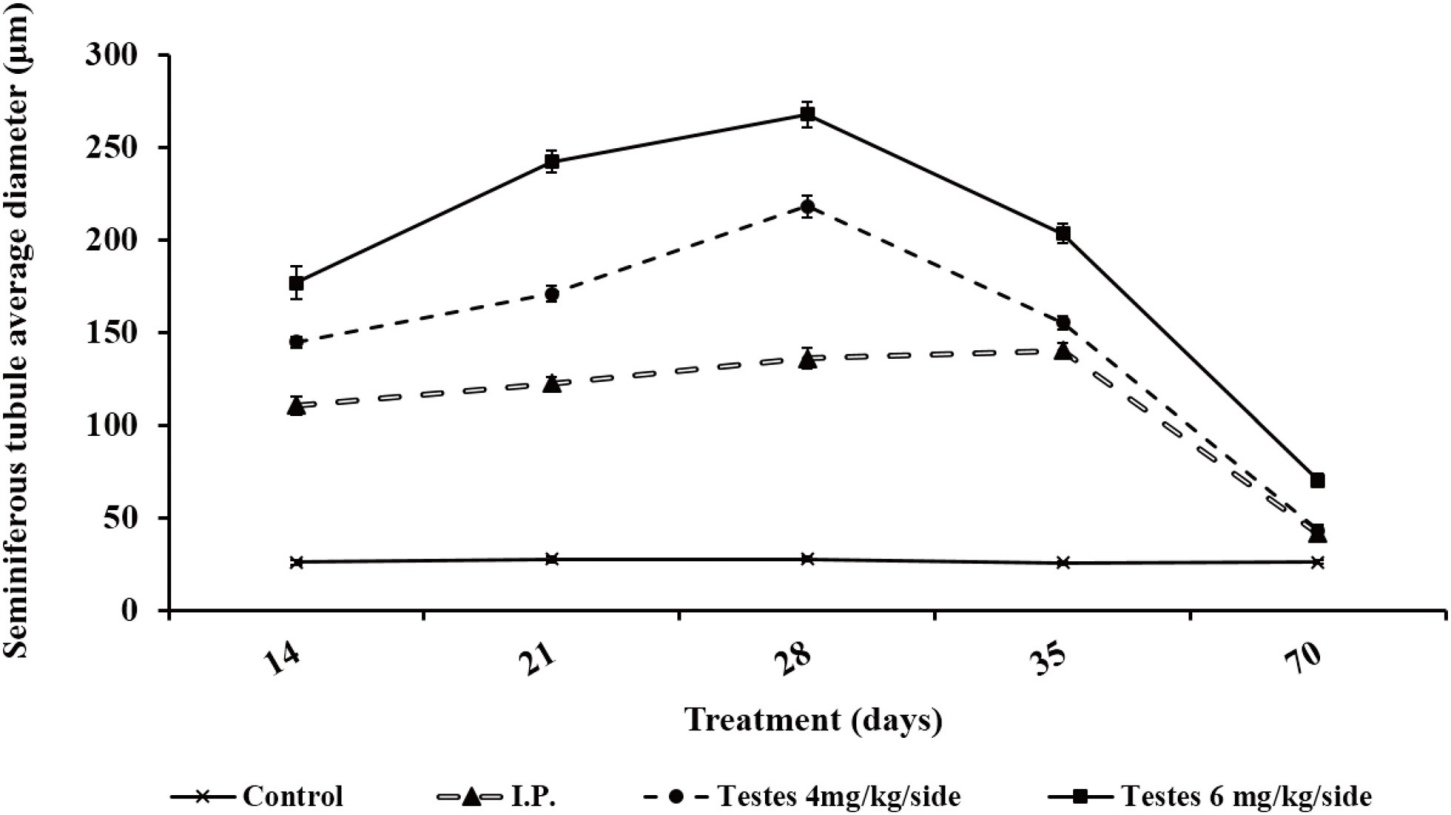
The cavity diameter of seminiferous tubules from mice administered with busulfan at the indicated routes and doses.

The average diameter of the seminiferous tubule cavities of mice in the testis-injection groups gradually increased (Figs 3 and 4, Table 2) and was significantly higher than that of those in the i.p. injection group after busulfan treatment (*P* < 0.05); it peaked on day 28 at 267.78 μm in the 6 mg/kg/side and at 218.01 μm in the 4 mg/kg/side, before gradually decreasing. There was significant difference in 6 mg/kg/side on day 70 after treatment compared with i.p. group (*P* < 0.05). Among these groups, the cavity hollows in the 6 mg/kg/side group appeared earlier (Fig 3B, 3C and 3D). The Sertoli cell only was observed clearly on day 14 after busulfan treatment and the largest hollow space appeared on day 21 and remained until day 28 (Figs 3C2 and 4), which was earlier than observed in the other groups.

**Table 2:** The Change of seminiferous tubules luminal diameter after different routes of administration.

Day after treatment	Group	Seminiferous tubules luminal diameter (μm)	Average diameter (μm)
14	Control	20.72–30.09	25.78±1.57^d^
	I.P.	98.45–128.64	110.53±5.07^c^
	Testes 4	142.27–152.16	144.71±2.97^b^
	Testes 6	150.38–203.22	176.77±8.99^a^
21	Control	23.59–31.22	27.47±1.37^d^
	I.P.	117.27–137.20	122.21±3.61^c^
	Testes 4	160.95–182.02	170.88±4.11^b^
	Testes 6	227.65–257.51	242.15±5.97^a^
28	Control	24.50–32.06	27.52±1.32^d^
	I.P.	92.56–113.62	135.95±5.80^c^
	Testes 4	200.72–237.47	218.01±5.91^b^
	Testes 6	244.77–285.46	267.78±7.01^a^
35	Control	22.45–28.37	25.54±1.07^d^
	I.P.	47.16–75.95	140.02±3.98^c^
	Testes 4	144.86–166.39	155.17±3.78^b^
	Testes 6	189.25–217.84	203.44±5.32^a^
70	Control	23.85–28.37	26.08±0.88^c^
	I.P.	28.66–43.67	41.40±4.12^b^
	Testes 4	39.02–52.08	43.12±3.06^b^
	Testes 6	60.78–80.51	70.19±3.40^a^

Testes 6, testis 6 mg/kg/side dose group; testes 4, testes 4 mg/kg/side dose group;I.P., intraperitoneal busulfan injection group; Control, negative control group. Different letters (a, b, c and d) within a column denote a statistically significant difference compared with i.p. injection group (P < 0.05).

### Routine blood tests of the recipient mice following different busulfan treatment

**Fig 5:**
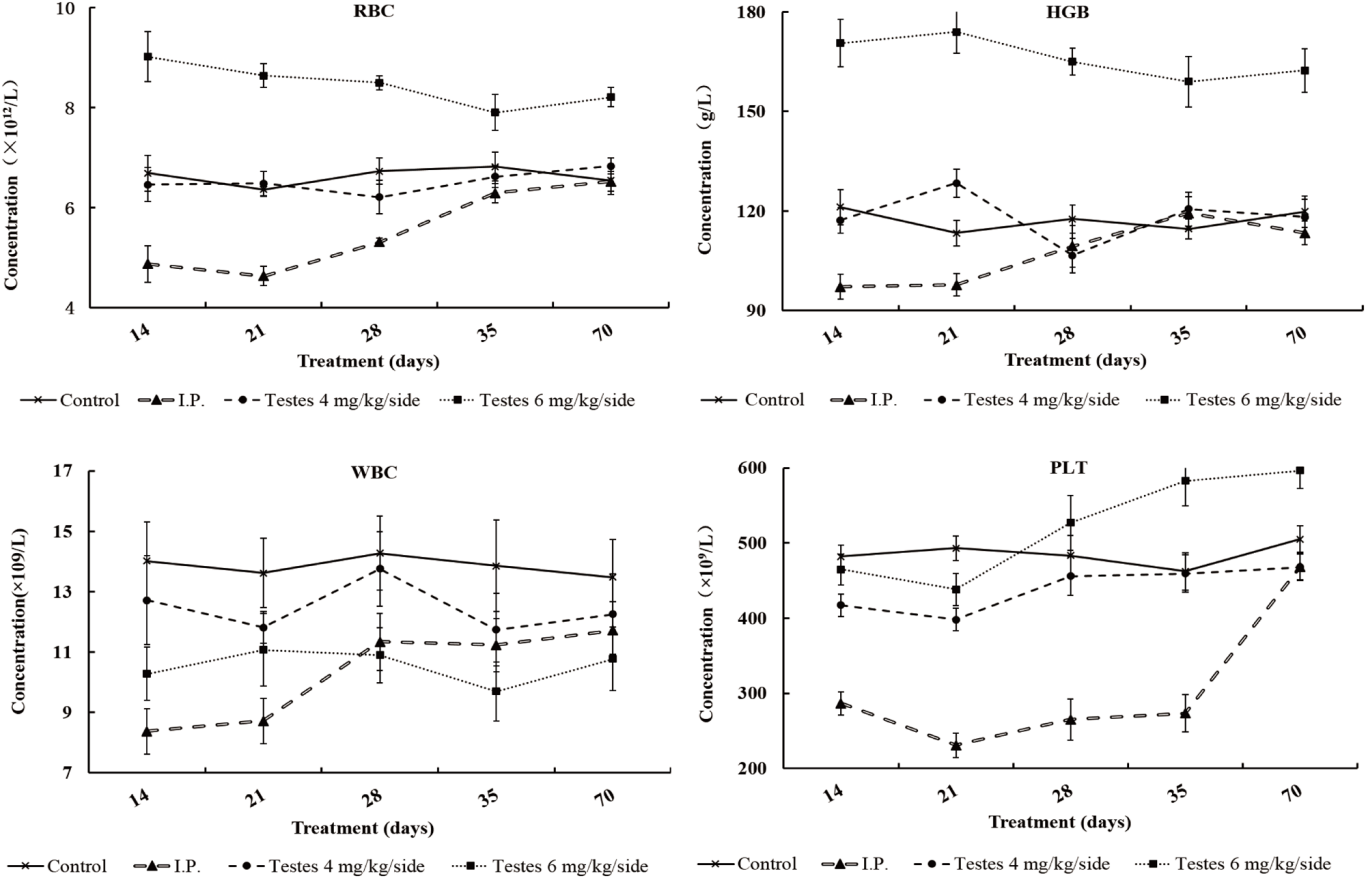
Changes in levels of WBC, RBC, HGB and PLT after busulfan treatment among groups. WBC, white blood count; RBC, red blood count; HGB, hemoglobin; PLT, platelets.

The RBC, WBC, HGB and PLT of the recipient mice were detected by a routine method at different times after busulfan treatment (Table 3). The values for RBC and HGB of mice in the i.p. injection group after busulfan treatment were significantly lower than those of mice in the control group on day 14 and day 21 (*P* < 0.05). Values in the i.p. injection group decreased immediately after budsulfan treatment and reached their lowest levels at day 21 (for RBC) and day 14 (for HGB), before gradually increasing until they were restored to normal levels on day 35 (for RBC) and day 28 (for HGB). There was no significant difference in RBC level between the 4 mg/kg/side dose group and control group (*P* > 0.05); however, the RBC levels of mice in the 6 mg/kg/side dose group were significantly higher than that of those in the other groups (including the control group) throughout the detection period (*P* < 0.05) (Fig 5, Table 3). The WBC and PLT levels were similar to the RBC levels in the i.p. group, as these were lower at day 14 and 21(for WBC) and the entire experiment time (for PLT) compared to mice in all other groups, before starting to increase slowly at day 14 (for WBC) and day 21 (for PLT). There was no significant difference for WBC values between the 4 mg/kg/side group and the control group, and there was also no significant difference for PLT values (*P* > 0.05), except for lower PLT values in the 4 mg/kg/side group on day 14 (*P* < 0.05). However, the WBC levels of the 6 mg/kg/side dose were significantly lower than that of mice in control group on days 28 and 35, but there were no significance between the i.p. group and 6 mg/kg/side group throughout the detection period (*P* > 0.05). There was only a slight decline of PLT in the 6 mg/kg/side dose group from day 14 to day 21, after which it steadily increased.

**Table 3:** The statistical results of blood indexes after busulfan treatment in different doses and administered methods.

Days after treatment	Group	WBC (10^9^/L)	RBC(10^12^/L)	HGB (g/L)	PLT(10^9^/L)
14	Control	14.02±1.29^a^	6.69±0.36^b^	121.20±5.27^b^	486.89±15.36^a^
	I.p.	8.38±0.75^b^	4.87±0.37^c^	97.20±3.71^c^	265.29±15.36^c^
	Testes 4	12.72±1.47^a^	6.46±0.34^b^	117.20±3.79^b^	451.84±14.72^b^
	Testes 6	10.28±0.88^ab^	9.02±0.50^a^	170.60±7.14^a^	474.16±20.61^ab^
21	Control	13.62±1.15^a^	6.36±0.14^b^	113.40±3.85^c^	517.33±16.13^a^
	I.p.	8.72±0.76^b^	4.63±0.19^c^	97.80±3.41^d^	204.22±16.13^c^
	Testes 4	11.82±0.52^a^	6.48±0.24^b^	128.40±4.30^b^	427.80±15.30^b^
	Testes 6	11.08±1.20^ab^	8.64±0.24^a^	174.00±6.44^a^	447.00±21.64^ab^
28	Control	14.28±1.22^a^	6.73±0.26^b^	117.60±4.18^b^	489.00±27.12^a^
	I.p.	11.34±0.95^ab^	5.31±0.08^c^	109.40±6.23^b^	295.67±27.12^b^
	Testes 4	13.76±1.23^ab^	6.21±0.33^b^	106.60±5.26^b^	484.80±25.73^a^
	Testes 6	10.90±0.91^b^	8.50±0.14^a^	165.00±4.09^a^	528.00±36.38^a^
35	Control	13.86±1.52^a^	6.82±0.29^b^	114.60±2.94^b^	443.11±25.00^b^
	I.p.	11.24±0.88^ab^	6.29±0.19^b^	119.40±4.97^b^	272.78±25.00^c^
	Testes 4	11.74±1.20^ab^	6.62±0.22^b^	120.60±5.06^b^	464.67±25.00^b^
	Testes 6	9.70±0.98^b^	7.90±0.36^a^	159.00±7.57^a^	584.40±33.53^a^
70	Control	13.48±1.26^a^	6.54±0.28^b^	119.80±4.66^b^	491.67±17.73^a^
	I.p.	11.72±0.96^a^	6.52±0.20^b^	113.40±3.60^b^	487.11±17.73^a^
	Testes 4	12.26±1.34^a^	6.83±0.16^b^	118.20±5.35^b^	472.70±16.82^a^
	Testes 6	10.78±1.05^a^	8.21±0.19^a^	162.40±6.52^a^	333.40±23.79^b^

Testes 6, testis 6 mg/kg/side dose group; testes 4, testes 4 mg/kg/side dose group; I.P., intraperitoneal busulfan injection group; Control, negative control group. Different letters (a, b, and c) within a column denote a statistically significant difference compared with i.p. injection group (P < 0.05).

### The number and gender of the offspring after different busulfan treatment

**Fig 6:**
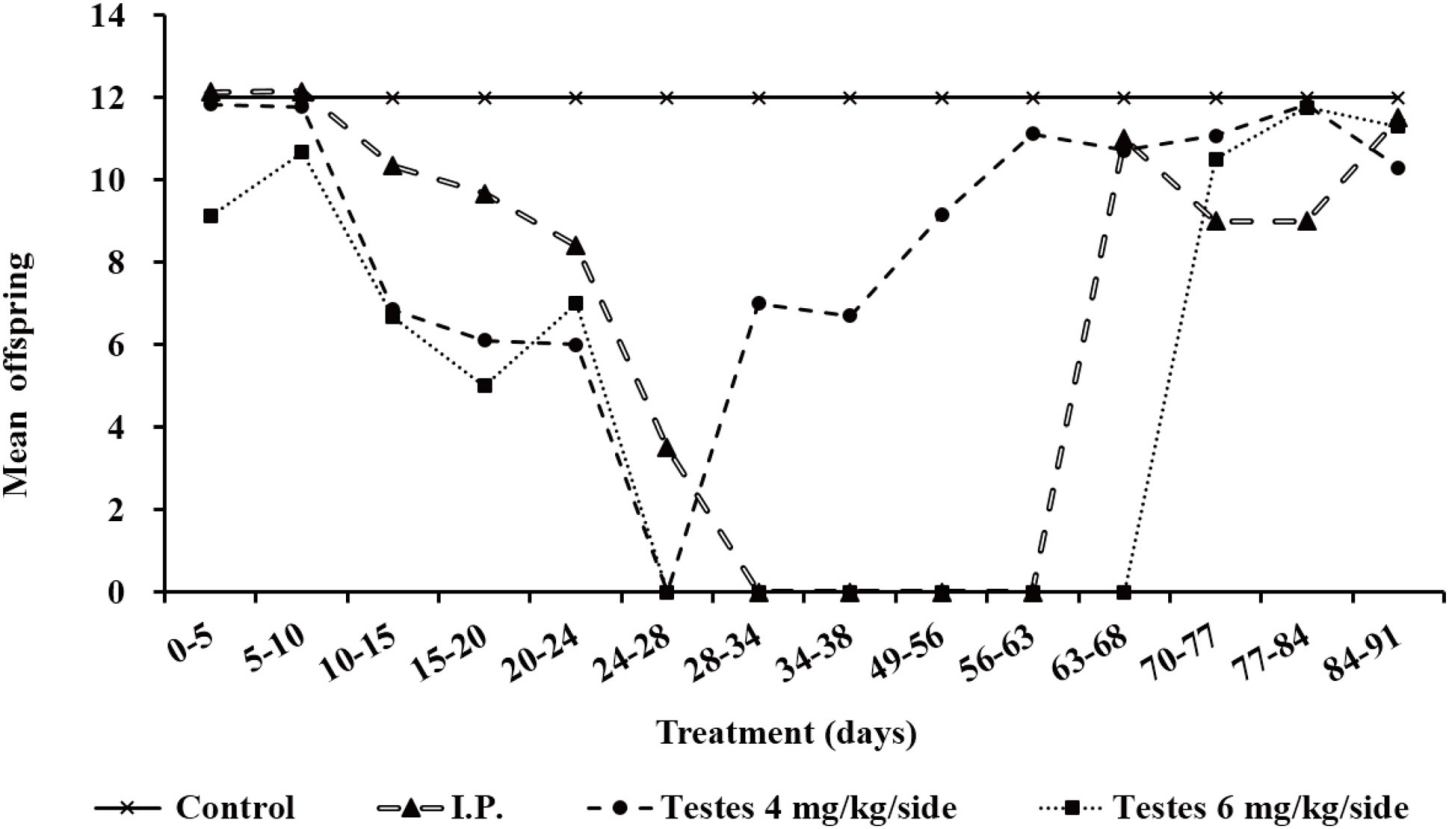
The number of offspring of the recipients treated with different doses of busulfan and different methods over time.

The fertility of the recipient mice in the different treatment groups was determined by natural service with ICR female mice after busulfan treatment. The results of the offspring produced showed that the fertility of recipient mice gradually decreased in all groups with time after busulfan treatment (Fig 6, Table 4). The fertility of mice in the testis injection groups decreased faster than that of those in the i.p. group: the fertility of mice in the testis injection groups was lost after day 24, until it began to restore at day 28 (in the 4 mg/kg/side dose group) and day 70 (in the 6 mg/kg/side dose group), whereas the fertility of mice in the i.p. group was lost after day 28 until day 63, when it began to restore (Fig 6, Table 4). Meanwhile, the changing trend of the pregnancy rate was similar with regards to the number of offspring. The sex ratios of the offspring in both the testes-injection and i.p. injection group was almost 1:1 (Table 4).

**Table 4:** The information of offspring produced by mice in each group after busulfan treatment.

Mating time after treatment/d	Group	No. of male	No. of Pregnant female	Pregnant rate (%)	Number of offspring	Ration of offspring (m:f)	Mean offspring of every mate
0–5	Testes 4	10	18	90	213	95:118	11.83 ^b^
	Testes 6	5	9	90	82	44:38	9.11 ^c^
	I.P.	5	8	80	97	45:52	12.13 ^a^
5–10	Testes 4	10	17	85	200	99:101	11.76 ^b^
	Testes 6	5	6	60	64	28:36	10.67 ^c^
	I.P.	5	7	70	85	42:43	12.14 ^a^
10–15	Testes 4	10	13	65	89	47:42	6.85 ^**b**^
	Testes 6	5	3	30	20	9:11	6.67 ^c^
	I.P.	5	6	60	62	31:31	10.33 ^a^
15–20	Testes 4	10	10	50	61	29:32	6.10 ^b^
	Testes 6	5	2	20	10	4:6	5.00 ^c^
	I.P.	5	6	60	58	32:26	9.67 ^a^
20–24	Testes 4	10	6	30	36	15:21	6.00 ^c^
	Testes 6	5	1	10	7	4:3	7.00 ^b^
	I.P.	5	5	50	42	22:20	8.40 ^a^
24–28	Testes 4	10	0	0	0	/	/
	Testes 6	5	0	0	0	/	/
	I.P.	5	2	20	7	3:4	3.50
28–34	Testes 4	10	5	25	35	19:16	7.00
	Testes 6	5	0	0	0	/	/
	I.P.	5	0	0	0	/	/
34–38	Testes 4	5	10	50	67	32:35	6.70
	Testes 6	5	0	0	0	/	/
	I.P.	5	0	0	0	/	/
49–56	Testes 4	10	14	70	128	68:60	9.14
	Testes 6	5	0	0	0	/	/
	I.P.	5	0	0	0	/	/
56–63	Testes 4	10	17	85	189	84:105	11.12
	Testes 6	5	0	0	0	/	/
	I.P.	5	0	0	0	/	/
63–68	Testes 4	10	17	85	182	79:103	10.71
	Testes 6	5	0	0	0	/	/
	I.P.	5	2	20	22	12:10	11.00
70–77	Testes 4	10	16	80	177	84:93	11.06 ^a^
	Testes 6	5	2	20	21	12:9	10.50 ^b^
	I.P.	5	2	20	18	11:7	9.00 ^c^
77–84	Testes 4	10	16	80	189	101:88	11.81 ^a^
	Testes 6	5	4	40	47	23:24	11.75 ^a^
	I.P.	5	1	10	9	6:3	9.00 ^b^
84–91	Testes 4	10	17	85	175	80:95	10.29 ^b^
	Testes 6	5	7	70	79	39:40	11.29 ^a^
	I.P.	5	4	40	46	20:26	11.50 ^a^
	Control	5	10	100	120	66:54	12.00

Testes 6, testis 6 mg/kg/side dose group; testes 4, testes 4 mg/kg/side dose group; I.P., intraperitoneal busulfan injection group; Control, negative control group. Different letters (a, b, and c) within a column denote a statistically significant difference compared with i.p. injection group (*P* < 0.05).

## Discussion

In this study, we found that the testicular injection of busulfan is a safe and feasible method to prepare recipients for SSC transplantation. Using this method, the toxicity to the hematopoietic system was found to be low, and the development of seminiferous tubule cavities was rapid. The transplant window is wide and mortality rates of recipient were zero [25]. This study is the first to demonstrate that recipients can be safely and viably prepared using the testicular injection of busulfan. Combined with xenograft technology, this could provide a feasible method for the further study of spermatogenesis and sperm development in humans using recipient animal models, such as the mouse or monkey. Such an application could be used to develop cures for male infertility and male contraception methods to avoid certain genetic diseases [6] and provide birth control [27]. With increasing rates of spermatogenesis errors [28], genetic diseases [29, 30]and male infertility [4, 31] studies that involve the xenotransplantation of SSCs will play an important role in this field [32]. It has been shown previously that when recipients were prepared using an i.p. injection of busulfan, recipient fertility was restored and the donor genotype was passed to progeny from recipients, or donor sperm were observed in the seminiferous lumens of the recipients after SSC transplantation [13, 17]. However, busulfan causes damage to the hematopoiesis function and severe side effects in rodents, when administered by an i.p. injection. Moreover, when the dose of busulfan exceeds 28 mg/kg by i.p. injection, the fertility of the recipients is not restored [14]. Methods of preparing the recipient testes by irradiation [33], and heat shock [24] have also been explored. Although these methods avoided the toxicity of busulfan, they did not overcome shortcomings such as a narrow transplant window, high cost, and side-effects. In this study, using a testis injection for recipient preparation, we have successfully obtained donor offspring and there were no recipient deaths. An i.p. injection of busulfan at a dose of 50 mg/kg is lethal for BALB/c mice, which is probably because of the cytotoxic effects on the hematopoietic system [9] and is supported by another study in which there was an obvious decrease in the survival rate when doses higher than 30 mg/kg were used in BALB/c mice [34]. A study in RAG2 mice also demonstrated an increased mortality rate with a busulfan dose increase from 20 to 40 mg/kg [35]; whereas in ICR mice, only one mouse died at a dose of 40 mg/kg [36]. In previous study, we found a higher mortality rate up to 31.6% [25] among ICR mice treated with 40 mg/kg busulfan by i.p. injection; however, the most important point is that no recipient mice died as a result of the testis busulfan injection. In previous studies, each recipient mouse was administered between 0.6–1.2 mg of busulfan [13, 35]. In this study, each mouse was only administered 0.36 mg of busulfan in our 6 mg/kg/side group, which was a decrease of 40%-70% on previous reports, thereby decreasing the toxic effects. Moreover, the direct injection into the testis markedly decreased the amount of busulfan that entered the vascular circulation, therefore avoiding the high mortality rate of recipient found previously among mice treated by i.p. injection.

Our study showed that the levels of RBC and HGB were higher in the 6 mg/kg/side a testis injection of busulfan dose group than in the other groups, and were always at a relatively steady state. However, a previous study that showed that the levels of RBC and HGB were significantly reduced to 62.1% and 65%, respectively, of the control group on day 1, when female CD-1 mice were treated with 9.0 mg/kg busulfan [37]. We think that the high levels of RBC and HGB in our study may be due to a strong compensatory response caused by the adverse stimuli of the testis injection. However, we did not detect the initial low level of RBC and HGB and their initially fine variation tendency because we did not start our sampling until day 14. Therefore, further studies are necessary to illuminate the variation tendency of RBC and HGB at earlier time points and at shorter intervals after busulfan treatment. In our study, there was no significant difference in PLT levels at day 14 between the 6 mg/kg/side group and the control group, but the PLT value was significantly decreased in the i.p. group. Previous studies have also reported a significant decrease in PLT levels in the i.p. group [37]. The PLT count decreased by approximately 96% at day 14 compared with the count of the same rat at day 1 [38].

The low levels of RBC, PLT, WBC and HGB decreased the transporting ability of oxygen and carbon dioxide, and along with immunologic competence, ultimately led to the death of recipients. In contrast, the levels of RBC, PLT, WBC and HGB in the testis injection groups indicated that there was very little effect on the hematopoiesis function using this method. Taken together with the fact that no recipient mice died in the testis injection groups, our results show that the testis injection is a safer method for administering busulfan than the i.p. injection.

In mice treated with 6 mg/kg/side busulfan, the cavity hollow appeared earlier than in mice in other groups and the largest hollow space was observed at day 21 after treatment and was maintained more than a month [25], which was a longer time period than observed that in mice treated with 40 mg/kg i.p. Moreover, the donor-derived offspring were eventually obtained through transplantation between days 16–17 after busulfan treatment. In a previous study, the Sertoli cell only remained the largest between 4 and 8 weeks when 30 mg/kg of busulfan was administered to ICR recipient mice by i.p. injection [36]. However, in another study, the seminiferous tubules in the 30 mg/kg busulfan group had been nearly occupied with germ cells at day 56 [17]. Therefore, stability is a limiting factor when busulfan administered by i.p. injection is used for recipient preparation. In heat shock experiments, although the maximum Sertoli cell only was reached on day 14 after heat shock treatment, the spermatogenesis gradually recovered on day 18 [24], which means that there was a narrow transplantation time window for SSCs transplantation. The testicular injection of busulfan also enables earlier transplantations to take place and a wider transplantation window is possible than with irradiation methods.

In conclusion, our study showed that only a slight toxic effect occurs with testicular injections of busulfan. The number of donor offspring showed that a testicular injection of busulfan is a feasible way of preparing the recipient for SSC transplantation. This method of recipient preparation opens up new avenues for the exploration of the mechanisms of human spermatogenesis and sperm development using animal models. Such models could be extensively applied in future studies on human reproduction, including those related to finding cures for male infertility, preventing the transmission of genetic disease and the development of male contraceptive methods.
